# Microvesicles from cerebrospinal fluid of patients with Alzheimer’s disease display reduced concentrations of tau and APP protein

**DOI:** 10.1038/s41598-019-43607-7

**Published:** 2019-05-08

**Authors:** Philipp Spitzer, Linda-Marie Mulzer, Timo Jan Oberstein, Luis Enrique Munoz, Piotr Lewczuk, Johannes Kornhuber, Martin Herrmann, Juan Manuel Maler

**Affiliations:** 1 Department of Psychiatry and Psychotherapy, Friedrich-Alexander-University Erlangen-Nuremberg, University Hospital Erlangen, Erlangen, Germany; 2 Department of Internal Medicine 3 – Rheumatology and Immunology, Friedrich-Alexander-University Erlangen-Nürnberg, University Hospital Erlangen, Erlangen, Germany; 3 Department of Neurodegeneration Diagnostics, Medical University of Bialystok, Bialystok, Poland

**Keywords:** Cell death in the nervous system, Alzheimer's disease, Alzheimer's disease

## Abstract

Microvesicles are small membranous particles generated during cellular activation or stress. The analysis of the content and the surface of microvesicles allow conclusions about the cells they are originating from and the underlying pathology. Therefore, CSF microvesicles have been suggested to be promising targets to monitor the (etio)pathology of neurodegenerative diseases. Microvesicles in the CSF of 15 patients with Alzheimer’s disease and 15 controls were analyzed by flow cytometry regarding the levels of CD3, CD4, CD45, CD64, BACE1, Aβ, APP and tau. The results were replicated in a second cohort comprising 14 patients with Alzheimer’s disease and 9 controls. The levels of tau and APP were reduced in microvesicles of Alzheimer’s disease patients. A significant change was neither observed in the number of microvesicles nor in the expression of the other antigens. Tau and APP in microvesicles separated patients with Alzheimer’s disease from controls with an AUC of 0.84 and 0.89 respectively. We conclude that tau and APP in CSF microvesicles are promising biomarkers which could directly provide information about the Alzheimer pathology on a cellular level.

## Introduction

Extracellular vesicles are a heterogeneous group of cell derived, membrane coated particles^1^. While a clear cut definition is hardly possible, extracellular vesicles are divided into exosomes, microvesicles and apoptotic bodies^2^. With a size between 30–100 nm, exosomes are the smallest particles. They are derived from the endosomal system and are released via fusion of multivesicular bodies with the cell membrane^1^. Apoptotic bodies form during apoptosis and are above 1000 nm in size. Microvesicles measure between 100–1000 nm and are created during cellular activation or under stress by a regulated external budding of the plasma membrane. During this process, phosphatidylserine is translocated from the inner to the outer leaflet of the lipid bilayer, where it can be detected by Annexin V (AxV). Microvesicles contain proteins and RNA of the cytoplasm and carry membrane antigens of their host cells on their surfaces^3^.

It was originally assumed, that the main function of microvesicles is the elimination of toxic metabolites from the cell^4^. Today several other physiologic functions are known, such as communication between cells, transfer of proteins, RNA, and receptors between cells, regulation of synaptic activity as well as immune modulation^3,5^. Also, cyto-protective properties of microvesicles have been repeatedly shown^6,7^.

As their generation is associated with cellular activation and stress, microvesicles are considered to be suitable biomarkers for several diseases. Carrying surface receptors and intracellular proteins of their originating cells, microvesicles can be regarded as reporters of the focus and kind of pathology^8^. While there are a lot of reports on changes in extracellular vesicle composition in stroke, neuroinflammation, epilepsy or brain tumors, little is known about their role in neurodegenerative diseases such as Alzheimer’s disease^9–12^. Unfortunately, most studies do not clearly distinguish between exosomes, microvesicles and apoptotic bodies^8^. In addition, almost nothing is known about extracellular vesicles derived from neurons.

Biomarkers are intended to portray aspects of the pathophysiology of diseases^13^. While the cause of AD is still under debate, the neuropathology is characterized by the deposition of amyloid-β (Aβ) fibrils and hyperphosphorylated tau, accompanied by neuroinflammation^14^. Increasing attention is being paid to the role of extracellular vesicles in this process^15^. It seems that oligomeric tau and Aβ can be transferred between cells via exosomes^16,17^. This gave rise to the hypothesis that extracellular vesicles may be the carrier of prion-like misfolded proteins spreading the disease^18^. Contrarily, there are also studies showing a degradation of fibrils in the presence of extracellular vesicles^19,20^.

Established biomarkers of AD have several shortcomings. Measurement is hard to standardize, they are expensive, require lumbar puncture or are associated with radioactivity^21^. The development of disease modifying therapies is especially impaired by the lack of biomarkers that indicate the activity of the disease and predict the rate of cognitive decline^22^. Therefore, the identification of such biomarkers is of special interest^23^.

This study aims to detect differences in the composition of microvesicles as well as their load with tau and Aβ peptides in the cerebrospinal fluid (CSF) of patients with AD.

## Material and Methods

### Patients

The study protocol was approved by the ethic committee of the University Hospital Erlangen-Nuremberg (Nr. 3987), carried out according to the ethical guidelines of the Declaration of Helsinki and patients or their legal guardians provided their informed written consent.

Patients included in this study were referred to our memory clinic due to cognitive complaints. They underwent a psychiatric, neurological, medical and routine laboratory examination. Cognitive deficits were evaluated using the CERADplus battery^24^. An MRI scan was performed to evaluate brain atrophy and rule out vascular disease. The diagnosis of AD was made according to the NINCDS-ADRDA criteria taking into account the Aβ_42_/Aβ_40_-ratio, tau and phospho-tau in CSF^25,26^. The results of the diagnostic CSF biomarkers were interpreted according to the Erlangen Score (ES) algorithm^27^. Exclusively, either patients with dementia or MCI with high evidence of AD pathophysiological process (Alzheimer’s disease group, Erlangen Score ≥3) or without evidence of AD pathophysiological process (Control, Erlangen Score ≤1) were included in this study. In reference to the criteria suggested by Jack *et al*., the patients within the AD group are positive for amyloid and tau pathology as well as neurodegeneration (A+/T+/N+) whereas those in the control group are A-/T-/N-^28^. Patients with intermediate signs of AD pathophysiology were excluded. Samples analyzed within the replication cohort were recruited independently according to the same procedure but one year later than the initial cohort.

### Isolation and storage of microvesicles

After the lumbar puncture, CSF was centrifuged (750 g/5 min) within 30 min. The supernatant containing the microvesicles was collected, aliquoted and stored at −80 °C until analysis. For the evaluation of the impact of centrifugation and storage temperature on the microvesicles, these parameters were varied as indicated.

### Flow cytometry

The CSF containing the microvesicles was thawed and immediately incubated with human polyvalent immunoglobulin (Beriglobin, CSL Behring, Marburg, Germany) for 25 min at room temperature (RT) to avoid unspecific binding of antibodies. AxV-FITC (Immunotools, Friesoythe, Germany) and FM4-64 (ThermoFischer scientific, Waltham, MA, USA) were used to define the microvesicle population. Anti CD3-FITC (clone: UCHT-1), CD4-FITC (clone: EDU-2), CD45-FITC (clone: MEM-28) (all Immunotools, Friesoythe, Germany), CD64-FITC (clone: 10.1) (BD Pharmingen, Heidelberg, Germany), anti Aβ-AF488 (clone 6E10, BioLegend formerly Covance, San Diego, CA, USA), anti tau-1-FITC (clone PC1C6), anti amyloid precursor protein (APP)-AF488 (clone 22C11) (both Merck-Millipore, Darmstadt, Germany) or polyclonal anti beta site APP cleaving enzyme 1 (BACE-1) FITC (Sino Biological, Beijing, China) were added and incubated at 4 °C for 30 min. As recommended, unlabeled samples served to determine the total number of microparticles and the background fluorescence^29^. Fluorescent positive events were defined by their differentiation from antigen-negative-events in stained samples^30^. Antibodies of the respective immunoglobuline isotype served as additional controls. Samples were measured undiluted with a Gallios flow cytometer (Beckman Coulter, Brea, CA, USA). Excitation for fluorescence was at 488 nm and emitted fluorescence was recorded on the FL1 sensor (525/38 nm BP) as the AUC of FL1. Data analysis was performed with Kaluza software version 1.5 (Beckman Coulter, Brea, CA, USA).

### Statistical analysis

Statistical analysis was carried out with Prism 6.0 (GraphPad Software Inc., La Jolla, CA, USA). As not all datasets followed a Gaussian distribution, the non-parametric, two-sided Mann Whitney U Test was applicated. In the replication cohort outliers were removed with the ROUT method (Q = 1%). Correlations were calculated with the Spearman-Test. To evaluate the diagnostic accuracy, receiver operating curves (ROC) were used. Results are presented as median with interquartile ranges and were considered to be significant at a p-value < 0.05.

### Ethics approval and consent to participate

The study protocol was approved by the ethic committee of the University Hospital Erlangen-Nuremberg (Nr. 3987) and patients or their legal guardians provided their informed written consent.

## Results

### Study population

Patients were recruited at the memory clinic of the department of psychiatry, University Hospital Erlangen. Only those with a conclusive clinical and biomarker workup were included in the study. 15 patients were included in the AD group. All displayed an amnestic phenotype in the neuropsychological tests, suffered from a continuous cognitive decline and showed no clinical or biomarker signs of other neurodegenerative diseases. Neurochemical dementia diagnostics using the Aβ ratio, tau and p-tau were positive for all three biomarkers in each patient. In respect to disease severity, nine patients were in the stage of mild cognitive impairment, while six fulfilled the clinical criteria of mild dementia. The control group consisted of 15 patients with cognitive complaints due to depression, Parkinson’s Disease, frontotemporal lobe degeneration or vasculopathy. Neurochemical dementia diagnostics were negative for all three biomarkers for each patient (Table 1). Due to the prospective collection of samples, the age of the included patients could not be properly matched, resulting in a significant higher median age in the AD group. One patient in the control group suffered from a cold. Several others had a clinically non-significant elevation of the CRP resulting in an overall increase of CRP in the control group. In the replication cohort, consisting of 14 patients with Alzheimer’s disease (5 with MCI due to AD) and 9 controls, selected in the same way, as the initial cohort, the median age in the AD group was also about ten years higher.

**Table 1:** Patient characteristics.

N (female)	CON		AD	
	15	(4)	15	(8)
Age	59.2	[49.2–73.7]	74.3	[65.2–79.2]**
MMSE	30.0	[28.0–30.0]	26.0	[21.8–28.3]***
CDT	1.0	[1.0–2.0]	3.0	[1.0–3.0]*
CRP [mg/dl]	2.90	[1.90–8.90]	1.20	[0.58–2.30]**
CSF Aβ-ratio	0.075	[0.069–0.079]	0.034	[0.029–0.040]***
CSF t-tau [pg/ml]	241	[189–273]	535	[469–866]***
CSF p-tau [pg/ml]	42.4	[34.2–52.4]	89.5	[76.9–107.0]***
CSF Erythrocytes [/µl]	0.0	[0.0–1.0]	1.0	[0.0–70]

Patient characteristics are presented as median [interquartile range]. CON: control, AD Alzheimer’s disease, MMSE: mini mental status examination; CDT: clock drawing test; CRP: C-reactive protein; CSF: cerebrospinal fluid *p < 0.05, **p < 0.01, ***p < 0.001.

### No change in the total number of microvesicles in the CSF of AD patients

**Figure 1:**
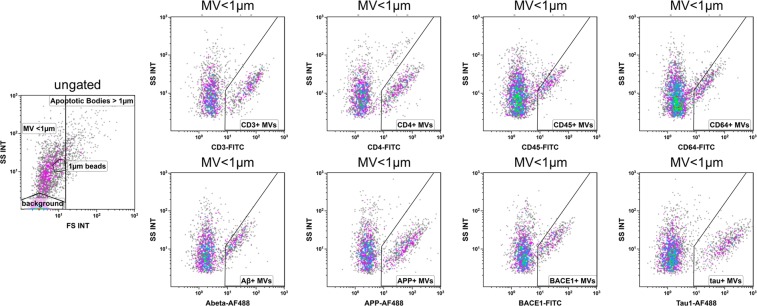
Gating of microvesicles. The respective antibody was added to CSF, incubated and measured directly and undiluted with flow cytometry. Microvesicles were defined by a gate that excluded background and particles with a higher forward scatter than 1 µm beads. The expression of CD3, CD4, CD45, CD64, Aβ, APP, BACE1, and tau was analyzed within the microvesicle gate (MV < 1 µm). Gates defining the respective antigen positive fraction were drawn in respect to an obviously antigen negative fraction within the same measurement.

**Figure 2:**
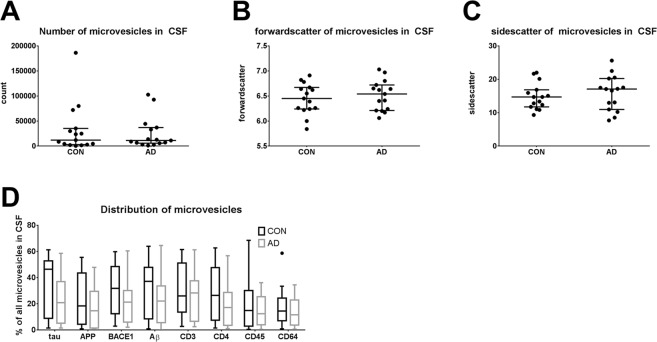
Number and shape of microvesicles in AD. The number of microvesicles (**A**), their forward scatter (**B**), and their side scatter (**C**) was analyzed within the MV < 1 µm gate as defined in Fig. 1 in unstained samples. No change was observed between patients with Alzheimer’s disease (AD) and patients with cognitive complaints but without AD (CON). (**D**) Microvesicles in the CSF of 15 AD patients and 15 controls were gated as shown in Fig. 1. The fraction of microvesicles staining positive for the respective antigen is depicted in a tukey-plot. No significant differences were observed between AD patients and controls.

In this sample consisting of 15 patients with AD and 15 controls, microvesicles in CSF were quantified by flow cytometry according to the gating strategy displayed in Fig. 1 (Fig. 1). According to forward- and sidescatter characteristics a gate was defined to include events that were clearly distinct from background but had a smaller forwardscatter than 1 µm polystyrene beads. Microvesicle counts were quantified in unstained samples (without the addition of antibodies). The number of microvesicles in CSF did not differ between controls and AD patients (Fig. 2). Also, size and structure of the microvesicles, as determined by forward- and sidescatter characteristics, did not differ between the groups (Fig. 2).

### Composition and characterization of CSF microvesicles

Single color staining was performed to measure APP, Aβ, BACE-1, tau, CD3, CD4, CD45, and CD64 to determine the origin of the microvesicles. Within the microvesicle gate defined above, the fraction staining positive for the respective antibody was defined in comparison with a clearly distinguishable unstained population within the same measurement (Fig. 1). For intracellular antigens, the permeability of the microvesicles for the antibodies was confirmed with an α-actin antibody staining. As events within the microvesicle gate were shown to be over 80% FM4-64 and AxV positive, these stainings were not included in each measurement to avoid steric interference and fluorescent quenching (Supplementary Fig. 1).

In the 15 control samples, the most abundant fraction of microvesicles was tau-positive (30.9% [16.8–52.5]) followed by the APP-positive (26.5% [4.3–49.7]), the Aβ-positive (20.1% [6.24–45.0]) and the CD3-positive (18.3 [7.79–45.9]) population (Fig. 2D). No change in the proportion of different microvesicles was observed in AD (Fig. 2D).

For the number of microvesicles, tau, APP, Aβ, CD3, and CD4 the respective inter-and intra-assay coefficients of variation (CV) were calculated by either measuring one sample repeatedly on five consecutive days or within one experiment (Table 2). While the intraassay CV was acceptably low and exceeded 20% only for CD3, the interassay variations were high. Normalization to background did not lead to more homogeneous results. To obtain comparable data, all samples in this study were measured in a single experiment. Inter-aliquot coefficients of variation were calculated by comparing results of five different aliquots measured in one assay. The inter-aliquot CV was highest for Aβ (41.3%) and lowest for CD4 (6.7%). For tau the inter-aliquot CV was 12.0%.

**Table 2:** Intra-, interassay and inter-aliquot coefficients of variation.

	Intraassay CV	Interassay CV	Interassay CV	Inter-Alliquot CV
		without background normalization	with background normalization	
replications	5	5	5	5
number MV	2.1%	42.3%	n.d.	24.5%
Tau (MFI)	15.4%	7.3%	28.03%	12.0%
APP (MFI)	3.3%	26.0%	33.09%	22.6%
Aβ (MFI)	9.4%	125.2%	128.89%	41.3%
CD3 (MFI)	4.5%	18.6%	30.8%	25.8%
CD4 (MFI)	4.2%	28.5%	39.82%	6.7%

CV: coefficient of variation, MV: microvesicles; MFI: mean fluorescent intensity.

### Microvesicles from cerebrospinal fluid of patients with Alzheimer’s disease contain reduced amounts of tau protein

**Figure 3:**
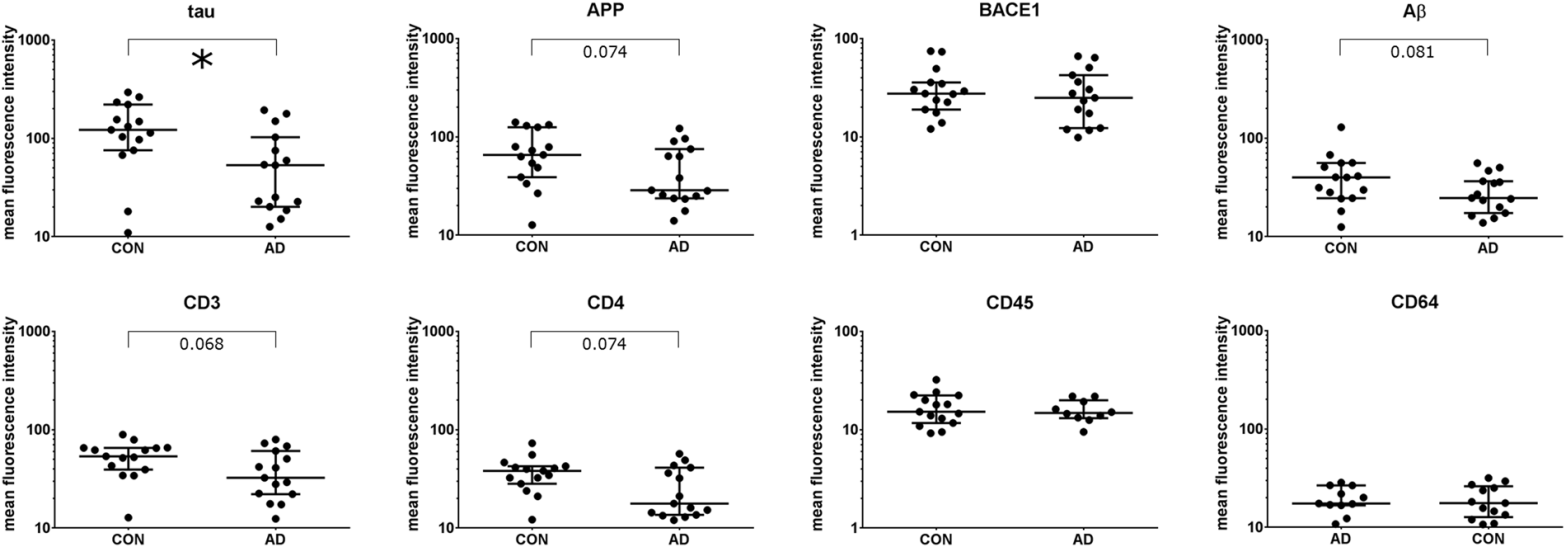
Reduced tau in CSF-derived microvesicles in AD. Microvesicles in the CSF of 15 AD patients and 15 controls were gated as shown in Fig. 1. The mean fluorescence intensities (MFI) for CD3+, CD4+, CD45+, CD64+, Aβ+, APP+, BACE1+, and tau+ microvesicles are shown for AD patients and controls. Differences between the groups were calculated with the non-parametric Mann-Whitney U Test. *p < 0.05.

While the number of tau positive microvesicles did not vary between the groups, the mean fluorescent intensity (MFI) of tau-AF488 in tau positive microvesicles was significantly lower in AD patients (Fig. 3). When normalized to unstained microvesicles, or to microvesicles stained with an isotype control, this difference remained significant.

The MFI of the APP positive microvesicles was lower in AD patients without reaching the level of significance (Fig. 3). As for tau positive microvesicles, no change in the number of APP positive microvesicles was observed. Normalization to the fluorescence intensity of unstained microvesicles or the isotype-control did not lead to different results.

Neither for the MFI of APP positive microvesicles nor for the MFI of tau positive microvesicles correlations with age or the concentrations of Aβ_1–40_, Aβ_1–42_ and tau in CSF were found. Also, the MFIs did not correlate with the number of microvesicles. There was no association with the MMSE. However, a highly significant correlation was found between the MFI of tau and APP in the microvesicles of AD patients (r = 0,88; p < 0.0001).

While no statistically significant difference in the number or fraction of Aβ, BACE1, CD3, CD4, CD45, and CD64 positive microvesicles was found, there was a trend towards a lower MFI of CD3 and CD4 in AD (Fig. 3).

### Tau in CSF microvesicles as a biomarker for AD

**Figure 4:**
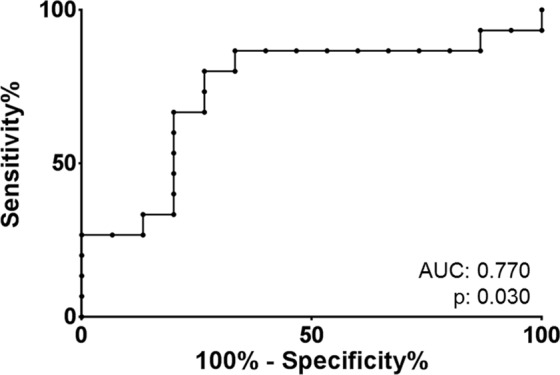
Receiver operating characteristics for tau in microvesicles. Receiver operating characteristics (ROC) were calculated for the differentiation of AD patients from controls by the mean fluorescence intensity (MFI) of tau in tau positive microvesicles.

To test whether tau in microvesicles might be a suitable biomarker for AD, receiver operating characteristic (ROC) curves were calculated. Within our sample we found an accuracy of 0.77 (0.76 when normalized on background fluorescence) for the separation of patients with high evidence of AD pathology (n = 15) from those with no biomarker evidence of AD pathology (n = 15) (Fig. 4).

### Confirmation of reduced microvesicle tau and APP in a independent replication cohort

**Figure 5:**
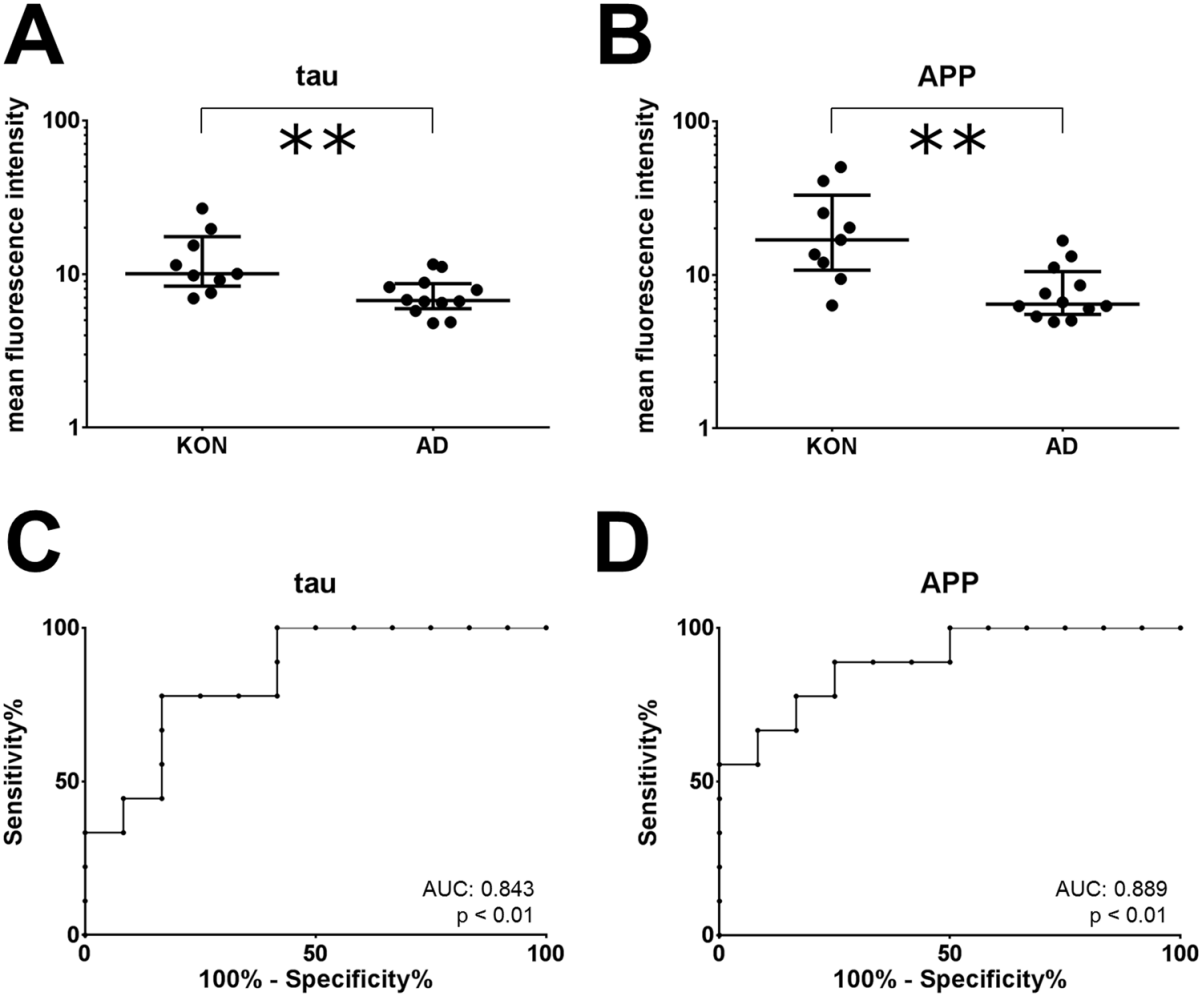
Replication of reduced tau and APP in CSF-derived microvesicles in AD. Microvesicles in the CSF of 14 AD patients and 9 controls were gated as shown in Fig. 1. The mean fluorescence intensities (MFI) for tau+ (**A**) and APP+ (**B**) microvesicles are shown for AD patients and controls. Differences between the groups were calculated with the non-parametric Mann-Whitney U Test. Receiver operating characteristics for both parameters are shown in (**C**) and (**D**). **p < 0.01.

The MFI of tau on microvesicles was also reduced in the second, independent replication cohort (Table 3), (Fig. 5A). While there was only a trend for reduced microvesicle-borne APP in the initial cohort, this difference was significant in the replication study (Fig. 5B). As the laser of the flow cytometer had been replaced between the two studies, the measured values are lower in the replication study and can therefore not be combined with the first study. Outliers, identified by the ROUT method, were removed from the analysis. For tau, there were two outliers in the AD group with a measured MFI of 42.4 and 30.6. Also in the measurement of APP there were two outliers in the AD group with MFI values of 146.8 and 33.1. Without the removal of the outliers, the difference between the study groups is still significant for APP (p = 0.028) and is reduced to a trend (p = 0.083) for tau. Again, we found no correlation of microvesicle tau or APP with age, the established CSF biomarkers or with the MMSE.

**Table 3:** Patient characteristics in the replication study.

N (female)	CON		AD	
	9	(3)	14	(7)
Age	65.1	[54.3–70.63]	76.9	[75.0–80.2]***
MMSE	27.0	[23.3–28.6]	24.0	[22.0–26.75]
CDT	2.0	[1.0–4.0]	3.0	[1.75–3.0]
CRP [mg/dl]	1.40	[0.85–6.55]	0.75	[0.42–3.70]
CSF Aβ-ratio	0.083	[0.064–0.089]	0.036	[0.030–0.039]***
CSF t-tau [pg/ml]	197	[113–290]	878	[510–1298]***
CSF p-tau [pg/ml]	36.5	[24.3–46.2]	89.1	[74.3–141.4]**
CSF Erythrocytes [/µl]	1.0	[0.0–49.5]	0.5	[0.0–18.8]

Patient characteristics are presented as median [interquartile range]. CON: control, AD Alzheimer’s disease, MMSE: mini mental status examination; CDT: clock drawing test; CRP: C-reactive protein; CSF: cerebrospinal fluid *p < 0.05, **p < 0.01, ***p < 0.001.

A ROC analysis in the second cohort resulted in a AUC of 0.84 (p < 0.01) for tau and 0.89 (p < 0.01) for APP (Fig. 5C,D).

### Sampling and storage conditions

Optimized treatment and storage conditions were established prior to this study. Therefore, the total number of FM4-64+/AxV+ microvesicles were assessed by flow cytometry. Storing the uncentrifugated samples at 4 °C or −20 °C increased the number of microvesicles up to threefold. Centrifugation of CSF at 750 g/5 min within 30 min after sampling only marginally reduced the total number of microvesicles. Subsequent storage at RT, 4 °C or −20 °C for up to 72 h did not change the number of microvesicles relevantly (Supplementary Fig. 2). Therefore, the samples within this study were centrifugated at 750 g/5 min and stored frozen until the analysis.

## Discussion

This study detected a similar distribution of the counts of CSF-borne microvesicles from AD patients and controls. Microvesicles derived from CSF of patients with AD had a reduced concentration of tau and APP protein. The results were replicated in an independent cohort.

This is one of the first studies that describe changes in the composition of neuronal microvesicles in the context of AD. Due to a high cell turnover and activation in patients with AD we expected an elevation of the microvesicle count. However, the inter-assay and inter-aliquot variability was high and may have obscured these differences. Since the relatively small cohorts allowed only the detection of large differences, we were intrigued that the tau and APP content differed significantly between patients with AD and controls and concluded that this is a pretty robust biomarker for AD.

Another problem is the age-difference of more than ten years between the study groups in both cohorts. The difference occurred because the samples had to be collected prospectively at the time of the lumbar puncture, when the diagnosis was not yet known. A balanced matching was not possible afterwards. However, as no correlation of age with the measurement parameters was found, it seems unlikely that the age difference interfered with the results. Nonetheless, the results need validation in a larger, age matched cohort.

As high inter-assay coefficients of variations were also reported in other studies, the question arises if flow cytometry is an appropriate method to quantify microvesicles^31^. Much effort has been undertaken to answer that question and the discussion is still controversial. While difficulties in standardization, measuring at the lower limit of detection, differentiation from background, swarming and fluorescent cross talk are serious problems, flow cytometry is still the only method which can distinguish membrane coated vesicles from other particles, determine the origin of the vesicle and allow the analysis of their size and form at the same time^29^. We propose not to use the particle count but the antigen detection (e.g. of tau) as diagnostic marker.

In previous studies, especially the impacts of the choice of a triggering signal as well as preanalytical handling have been investigated^32^. Linear fluorescence as trigger event is not recommended due to the unfavorable ratio of particle size to laser beam. This way, large amounts of unbound dye are registered together with the particle leading to a high background fluorescence and weakening the signal to noise ratio. Even AxV, a dye with a very high affinity cannot fully differentiate a microvesicle from background^32^. Furthermore, it is even in doubt, if all microvesicles bind AxV^33,34^. As a consequence, an unbiased triggering by front scatter combined with bead-based definition of particle size is recommended^32^. The Beckman & Coulter Gallios flow cytometer, used in this study was previously described as a superior instrument for measuring microvesicles based on forward scatter^35^.

Sampling, centrifugation and storage were reported to be critical for correct microvesicle analysis^29,36^. As recommended, samples within this study were processed within 30 minutes after sampling, agitation was avoided and centrifugation was optimized. For the measurement of microvesicles in blood, it was previously shown that low centrifugation is essential for absolute quantification but impairs separation from background noise^37^. In accordance with our data, others reported that no centrifugation leads to fragmentation of cells during freezing, but too high centrifugation reduced microvesicle counts considerably^32,37^. However the compromise, an intermedium centrifugation speed, as performed in our study, may lead to a higher variability in microvesicle counts when samples are frozen^29^. This holds also true for our experiments with inter-aliquot coefficients of variation of up to 41.3%. Therefore, the high inter-assay variation may in great parts be explained by the high inter-aliquot variations.

As specified above, microvesicles are expected to have a plethora of functions. Therefore, one can only speculate on the pathophysiological implications of our findings. As can be concluded from the missing correlation between soluble tau in CSF and the tau in microvesicles, the two parameters reflect different aspects of the tau pathology in AD. For soluble tau it is broadly accepted that it is released into the CSF during neuronal injury^26,28^. As microvesicles contain the cytoplasm of their cells of origin, microvesicle tau is supposed to display changes of intracellular tau metabolism.

On one hand, the reduced tau immunofluorescence may indicate that the binding epitope of the antibody is masked by phosphorylation, agglutination or by binding to carrier proteins^38^. This would be of special interest in terms of the hypothesis that microvesicles may also spread prion-like proteins^39,40^.

On the other hand a reduced tau concentration in microvesicles may be explained by an altered distribution of intracellular proteins. Li and colleagues have shown that the phosphorylation of tau leads to reduced concentrations in the axonal compartment and its accumulation in the somato-dendritic compartment^41^. As synaptic activity includes the generation of microvesicles, reduced axonal tau may explain reduced microvesicle tau^5,42^. Also, an impaired sorting of tau into exosomes has been observed after mTor dysregulation typically found in AD^43^. The observation that tau found in exosomes from early AD patients is highly phosphorylated also hints towards a relation between AD, tau phosphorylation and its sorting to extracellular vesicles^44,45^. The reduced concentration of tau in microvesicles may therefore correspond to the intra-neuronal tau hyperphosphorylation and accumulation in AD.

Interestingly, others found decreased synaptic proteins in exosomes of AD patients^46^. Together with our observations that several other proteins also tended to be less prevalent in microvesicles of AD and that the reduced levels of tau were highly correlated with the reduced levels of APP, a general disruption of the sorting process of proteins into microvesicles can be postulated. Recently, a mass spectrometry study revealed reduced concentrations of proteins associated with the function of extracellular vesicles in AD brains^47^. The authors also hypothesized a disturbed cellular clearance by a lack of function of extracellular vesicles.

Apart from the pathophysiological implications, the question was whether CSF microvesicles could serve as a biomarker for AD. With an area under the curves (AUC) between of 0.77 and 0.89 in our study, this seems possible. An advantage of this study was that the control group did not consist of healthy individuals but of patients who also presented with memory complaints but were not diagnosed with AD. The AUC has therefore to be interpreted in the context of differential diagnosis, a field in which established biomarkers also loose diagnostic accuracy^48^. However, due to the high coefficients of variation associated with the methodology, improvements in standardization have to be achieved until a diagnostic application is possible.

While this is the first study which shows changes in neuronal microvesicles, others have already shown increased numbers of myeloid exosomes and microvesicles in AD^19,49^. Therefore, CSF microvesicles as biomarkers for AD seem to be a promising option which deserves further methodological developments. Especially a replication in a larger and age-matched sample is needed. It will be interesting to see, whether neuronal microvesicles can also be detected in blood as it was previously shown for exosomes^50^.

## Supplementary information

definition of microvesicles and optimization of preparation and storage conditions

## Data Availability

The datasets generated during and/or analysed during the current study are available from the corresponding author on reasonable request.
