# Supplementary material for: Microvesicles from cerebrospinal fluid of patients with Alzheimer’s disease display reduced concentrations of tau and APP protein
Source: Sci Rep. 2019 May 8;9:7089. doi: 10.1038/s41598-019-43607-7 (PMC6506501; doi:10.1038/s41598-019-43607-7)
Supplement: Supplementary file 1 — definition of microvesicles and optimization of preparation and storage conditions [file 41598_2019_43607_MOESM1_ESM.pdf]

Title:

Microvesicles from cerebrospinal fluid of patients with Alzheimer's disease display reduced concentrations of tau and APP protein

Authors:

Philipp Spitzer[1,*], Linda-Marie Runkel[1], Timo Jan Oberstein[1], Luis Enrique Munoz[2], Piotr Lewczuk[1,3], Johannes Kornhuber[1], Martin Herrmann[2,+], Juan Manuel Maler[1,+]

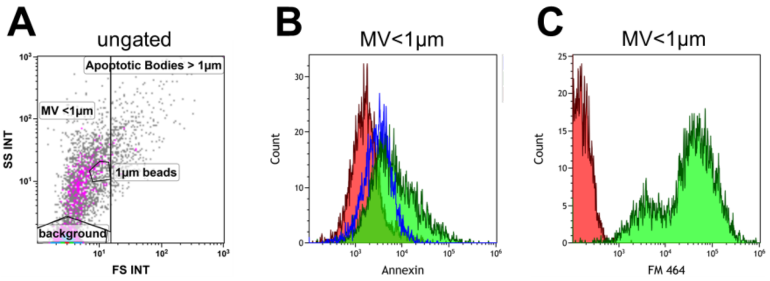

Supplementary Figure 1: Annexin V and FM4-64 staining of CSF-derived microvesicles

The microvesicle gate (MV<1µm) was defined to exclude background and particles with a higher forward scatter than 1µm beads (apoptotic bodies). Microvesicles were simultaneously labeled with annexin V (AxV)-FITC (B) and FM4-64 (C) to stain membranes. The red curves represent the background without the addition of the stain, whereas the green curve shows the mean fluorescence intensity of stained samples. The blue curve stem from AxV-FITC added together with EDTA, which avoids the binding of AxV to the microvesicles.

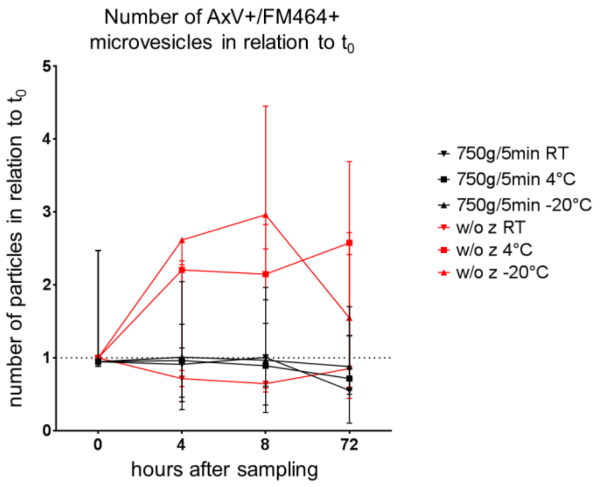

Supplementary Figure 2: Centrifugation and storage of CSF microvesicles

For an optimized preanalyical treatment of the samples, different centrifugation and storage protocols have been compared. The number of AxV and FM464 is displayed in relation to the uncentrifugated sample at t_0. The number of microvesicles increased when uncentrifugated samples were frozen. While centrifugation at 1500g and above resulted in a considerable loss of microvesicles (data not shown), the centrifugation at 750g / 5 min only resulted in a minor reduction in the number of microvesicles. The number of microvesicles remained constant during storage at different conditions when samples were centrifuged at 750g / 5 min.
